# mRNA splicing in turkey muscle satellite cells is dynamically altered upon thermal challenge

**DOI:** 10.1371/journal.pone.0349761

**Published:** 2026-05-27

**Authors:** Ashley A. Powell, Gale M. Strasburg, Sandra G. Velleman, Kent M. Reed

**Affiliations:** 1 Department of Veterinary and Biomedical Sciences, University of Minnesota, St. Paul, Minnesota, United States of America; 2 Department of Food Science and Human Nutrition, Michigan State University, East Lansing, Michigan, United States of America; 3 Department of Animal Sciences, The Ohio State University, Wooster, Ohio, United States of America; Karlsruhe Institute of Technology: Karlsruher Institut fur Technologie, GERMANY

## Abstract

Regulation of gene expression at the transcriptional and post-transcriptional levels is essential for proper development and growth, with tightly coordinated cellular processes supporting key biological functions. While transcription determines the available mRNA pool, post-transcriptional modifications such as alternative splicing (AS) increase transcriptome complexity and enable the production of diverse protein isoforms. In muscle, AS is critical in generating muscle-specific proteins required for normal development and function and may be particularly susceptible to disruption by thermal stress. This study examines how thermal challenges—both cold and heat—affect muscle biology by analyzing AS events during the proliferation and differentiation of skeletal muscle satellite cells (SCs). Isoform identification and AS analyses were performed on RNA-seq data from a prior study of skeletal muscle SCs derived from commercial turkeys and exposed to three temperature conditions (33°C, 38°C, or 43°C) during proliferation or differentiation. Analyses revealed 61,266 predicted splicing events across 5,202 annotated genes. Significant differential splicing was observed in all temperature comparisons, and between proliferating and differentiating cells at each temperature. Additionally, there was a strong association between differentially spliced genes (DASs) and differentially expressed genes (DEGs). This study provides a comprehensive catalog of splice isoforms for future functional analyses, many of which are likely to result in protein variants that influence SC proliferation, differentiation, and ultimately, muscle development and performance.

## Introduction

Climate change is of major concern to agricultural production as extreme temperatures, both cold and hot, coupled with high humidity negatively affect livestock, including poultry. Advances in genetic selection over the past five decades have significantly increased productivity and efficiency in poultry meat production. However, rapidly growing birds are more vulnerable to thermal stress, particularly heat stress [1], and increasingly display growth-associated myopathies. Thermal stress poses unique challenges for hatchlings and young birds before market age [2, 3].

Birds are homeotherms and the critical period in the development of thermoregulation occurs early after hatch when poults are especially susceptible to thermal extremes. In hatchlings, thermal stress changes muscle chemistry and structure resulting in increased lipid deposition and damage to fibers [4; 5]. These conditions affect muscle growth physiology and, consequently, final meat quality [6]. While heat stress presents greater physiological challenges than cold stress, exposure to hot or cold conditions can affect fundamental cellular processes including changes in the ratio of heat- and cold-sensitive cells in the brain, that modulate temperature tolerance [7].

Throughout development, cellular communication is essential for activating the genes needed for proper growth, with individual genes working together to support various biological functions [8]. Whereas the initiation of transcription is the primary control of gene expression, ultimate expression is also modulated by downstream processes. Beyond transcriptional regulation, processes like alternative splicing (AS) modify mRNA transcripts to enhance transcriptome diversity and generate a broader range of proteins with different structures, functions, and stabilities. In addition to increasing protein diversity, AS is a powerful regulatory mechanism that fundamentally changes the interactome of RNA molecules. By selectively including or excluding sequences (exons or introns), AS alters the physical properties of mRNAs (folding) and the landscape of mRNA interactions with other RNAs (microRNAs (miRNAs) or long non-coding RNAs (lncRNAs).

AS events defined by the changes made to the mRNA include skipped exons, retained introns, alternative 5’ or 3’ splice sites that exclude portions of exons, and alternative transcription start and stop sites [9]. Thus, AS is a key contributor to phenotypic complexity and results in the number of expressed proteins exceeding the number of individual genes, which plays a critical role in tissue-specific functions. AS is observed across eukaryotes, is estimated to impact 90–95% of human genes, and occurs more often than not [10]. Studies suggest that AS is regulated by multiple factors [11], including external temperatures [12], and is necessary for postnatal development [13, 14]. Studies in mice indicate that AS is vital for producing muscle-specific protein isoforms that are necessary for normal muscle function [14]. Patterns of AS are tissue-specific and disruptions in AS can lead to significant changes. For example, changes in AS in muscle can affect subsequent muscle structure and function [15].

While AS in the context of muscle development and function, has been studied in mammals, there is limited research in poultry. Development-specific AS events identified in chicken muscle highlight its potential role in growth and adaptation [16]. Most AS research in poultry has focused on chickens. Limited studies in turkey have examined genes of the Major Histocompatibility Complex [17] and the ryanodine receptor ion channel (RYR) genes in skeletal muscle. In muscle, altered expression of RYR splice variants were observed following heat stress with downstream effects on meat quality [18, 19].

Given the role of AS in muscle development, understanding its influence on muscle stem cells (satellite cells or SCs) is particularly important. SCs are responsible for post-hatch growth, as well as muscle repair and regeneration. Although muscle fiber formation is completed prior to hatch [20], post-hatch growth and muscle repair occurs through the process of hypertrophy. Muscle hypertrophy is dependent on the proliferation and differentiation of SCs [21] and maximal mitotic activity in poultry is observed during the first week post hatch [22; 23]. During this period, thermal stress (both cold and hot) alters gene expression during SC proliferation [24–26] and differentiation [26–28] and subsequently changes cellular fate [29]. The post-transcriptional molecular mechanisms that underlie thermal tolerance are still poorly understood.

During development, turkey skeletal muscle undergoes a cascade of programmed gene expression. Review of prior SC RNAseq studies [25, 28] found the expression of several genes with roles in RNA splicing affected by thermal challenge. Included in this set of genes are splicing regulators, splicing factors, serine and arginine-rich splicing factors (SRSF proteins) and spliceosome components. We hypothesized that alternative splicing of mRNA transcripts is an important component of the developmental gene expression cascade that is altered by thermal stress. This study investigates the effects of thermal challenge, cold and hot, on muscle biology by focusing on two main objectives: 1) Identify alternative splicing events in SCs during proliferation and differentiation; and 2) Test the impact of thermal stress on AS events. Here we utilize previously collected data to examine AS patterns, allowing for a deeper understanding of how thermal stress influences post-transcriptional regulation in turkey muscle development.

## Materials & Methods

### Materials

Transcript data (RNAseq) from commercial turkey SC libraries previously sequenced [25, 28] were used for alternative splicing analyses (NCBI SRA BioProject PRJNA842679). The SC cultures used for these studies were derived from a commercial meat-type turkey line (Nicholas Commercial Turkeys, NCT) selected for performance (yield, weight gain, feed conversion, etc.) in a modern turkey production system (Aviagen® Turkeys, Inc). Cultured cells from 1-week birds were subjected to proliferation and differentiation protocols to create two sets of SCs NCT-proliferation and NCT-differentiation used in the experimental treatments. The SC harvest, maintenance, differentiation and proliferation assays, and RNA sequencing were previously described [24,25,27,28,30]. Replicates of each SC set were subjected to three thermal treatments: 33°C (cold), 38°C (control), and 43°C (hot) resulting in 12 RNAseq libraries.

The reference turkey genome assembly (UMD5.1) and annotation from Ensembl (Meleagris_gallopavo.Turkey_5.1.dna.toplevel.fa genome; Meleagris_gallopavo.Turkey_5.1.109.gtf) were used for all analyses.

### Methods

#### Alternative splicing prediction.

Transcript quantifications were performed using Kallisto [31]. Isoform identification and AS analyses were performed in the IsoformSwitchAnalyzeR (ISA) package [32–37] in the R environment. Default parameter settings were used in R except when pre-filtering. The pre-filtering parameter was reduced to increase the number of genes analyzed. Code used can be found at https://github.com/reedx054-cmyk/ISA_Powelletal.

**Fig 1:**
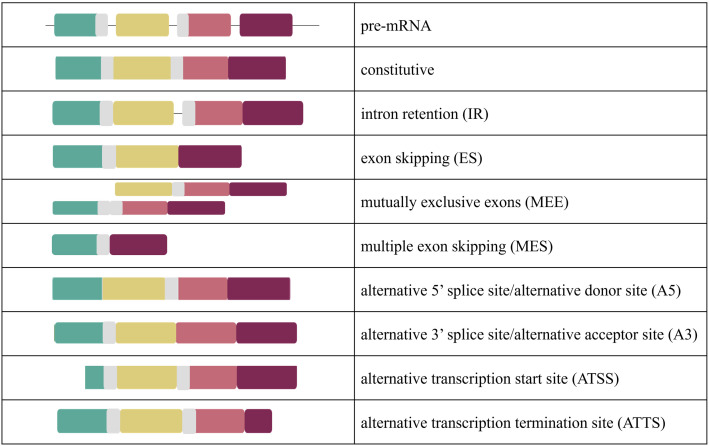
Illustration of the types of alternative splicing in the present study.

The ISA package identifies both alternative splicing events (ASE) and isoform switching events (ISE). For the sake of clarity and consistent language, we have defined the patterns of AS below (Fig 1). ASEs are defined as a single alternative splicing action (exon splicing (ES), intron retention (IR), etc) and ISEs are defined as all cumulative alternative splicing events (1+) that created a new isoform. Top isoform switching events (ISE) were generated with ISA ranked by |dIF|, or the absolute value of the difference of inclusion frequency of one isoform transcript in one condition (i.e., 33°C) vs another (i.e., 38°C). Comparisons were conducted between the thermal temperature treatments (33°C vs 38°C vs 43°C) in both proliferating and differentiating cells and between cell stages (proliferation vs differentiation) at the various temperatures.

### GO pathway analysis

Gene ontology and functional enrichment analyses were conducted using Panther19.0 (https://pantherdb.org/) and DAVID (https://davidbioinformatics.nih.gov/home.jsp). Results of each alternative splicing comparison were analyzed using the *Gallus gallus* gene set to identify gene pathways affected by splicing. Gene names were generated from the Ensembl gene ID output from ISA using the turkey gene sets in the Ensembl and NCBI databases. Sequences without ISA gene IDs were analyzed by BLAST analysis of the NCBI turkey RefSeq RNA database. DAVID analyses used the GOTERM_DIRECT and KEGG_Pathways options. Additional GO pathway analyses were run on genes with the highest |dIF| scores in the top 25 ISEs. A secondary GO pathway analysis was conducted with PANTHER to assess bias in directionality of splicing and expression. This analysis utilized genes differentially alternatively spliced (DASs) in the present study and differentially expressed (DEGs) found in the same dataset [25, 28].

### Secondary and tertiary structure predictions

To investigate potential functional effects of alternative splicing on secondary structure, an RNA folding prediction was conducted for select genes using RNAfold [Gruber et al., 2008] [38]  and the 2004 Turner Model [39]. The top 25 alternative splicing events per comparison were evaluated for genes that potentially had biological significance and had multiple isoforms within the RNAfold program limit (<7500 nt). Effects of AS on folding of isoforms at different temperatures were then predicted. Isoform nucleotide sequences predicted by ISA were used as input sequence and energy parameters rescaled to match treatment temperatures. miRNA interactions with isoform sequence were predicted with MiRanda 2.0 [40] with position-weighted scoring, alignment score >150 and |Energy-Kcal/Mol| > 7.0.

## Results

### Alternative splicing prediction

Five AS analyses of the RNAseq data were conducted, two among thermal treatments within cell stages and three between cell stages at the three experimental temperatures. A total of 61,266 splicing actions was predicted in the combined comparisons (Table 1). The predicted splice actions for 5,202 annotated genes (p_adj_ < 0.01) are summarized in Table S1 in S1 File. The number of AS events per gene ranged from 1 to 184 (average 11.58), with the majority of genes (4,907) having more than one type of AS event (average 3.73). Genes with the greatest number of AS events included *NCAM1* (neural cell adhesion molecule 1 or *CD56*), *R3HDM1* (R3h domain containing‐like, and *LOC100539553* (supervillin-like).

**Table 1:** Alternative splicing and isoform switch events identified in comparisons of RNAseq data from turkey muscle satellite cells.

	Control vs Cold vs Hot		Proliferation vs Differentiation		
Alternative Splicing Events	Proliferation	Differentiation	Control (38°C)	Cold (33°C)	Hot (43°C)
exon skipping (ES)	3227	3938	658	2430	577
mutually exclusive exons (MEE)	33	25	2	16	0
multiple exon skipping (MES)	812	1007	187	595	171
intron retention (IR)	1442	1532	283	1258	159
alternative donor site (A5)	2101	2489	394	1563	273
alternative acceptor site (A3)	3689	4197	647	2775	465
alternative transcription start site (ATSS)	3742	4254	682	2911	524
alternative transcription termination site (ATTS)	3832	4242	685	2927	522
Total alternative splicing events	18878	21684	3538	14475	2691
**Isoform Switching Events**					
Total isoform switching events (IS); p_adj_ > .01	7319	9735	1174	4563	903
Unique isoform switching events; p_adj_ > .01	6001	6793	1174	4563	903
Alternatively spliced genes; p_adj_ > .01	3167	3486	744	2607	574

Among the temperature treatments, the greatest number of AS events were observed for alternative transcription termination site (ATTS), alternative transcription start site (ATSS) and alternative acceptor site (A3) events, followed by exon skipping (ES), alternative donor site (A5), intron retention (IR), multiple exon skipping (MES) and mutually exclusive exons (MEE). With the exception of mutually exclusive exons (MEE), the number of AS events was greater in the differentiating cells. Differences in AS events between cell stages were greatest in the cold-treated cells (Table 1).

Comparison of the AS events found a total of 23,694 isoform switching (IS) events (p < 0.01) among the treatment groups (Table S2 in S1 File). The 10 genes with the greatest [dIF] scores across all comparisons were *PDE7B* (phosphodiesterase 7B), *XPO4* (exportin 4), *TMEM171* (transmembrane protein 171), *PTGES3* (prostaglandin E synthase 3), *ENSMGAG00000004026* (chromodomain Y like), *BACE2* (beta-site APP-cleaving enzyme 2), *ENDOD1* (endonuclease domain containing 1), *BLTP3B* (Bridge-Like Lipid Transfer Protein Family Member 3B), *ENSMGAG00000017949* (novel gene), and *FOXS1* (Forkhead Box S1).

### Thermal temperature comparisons

**Fig 2:**
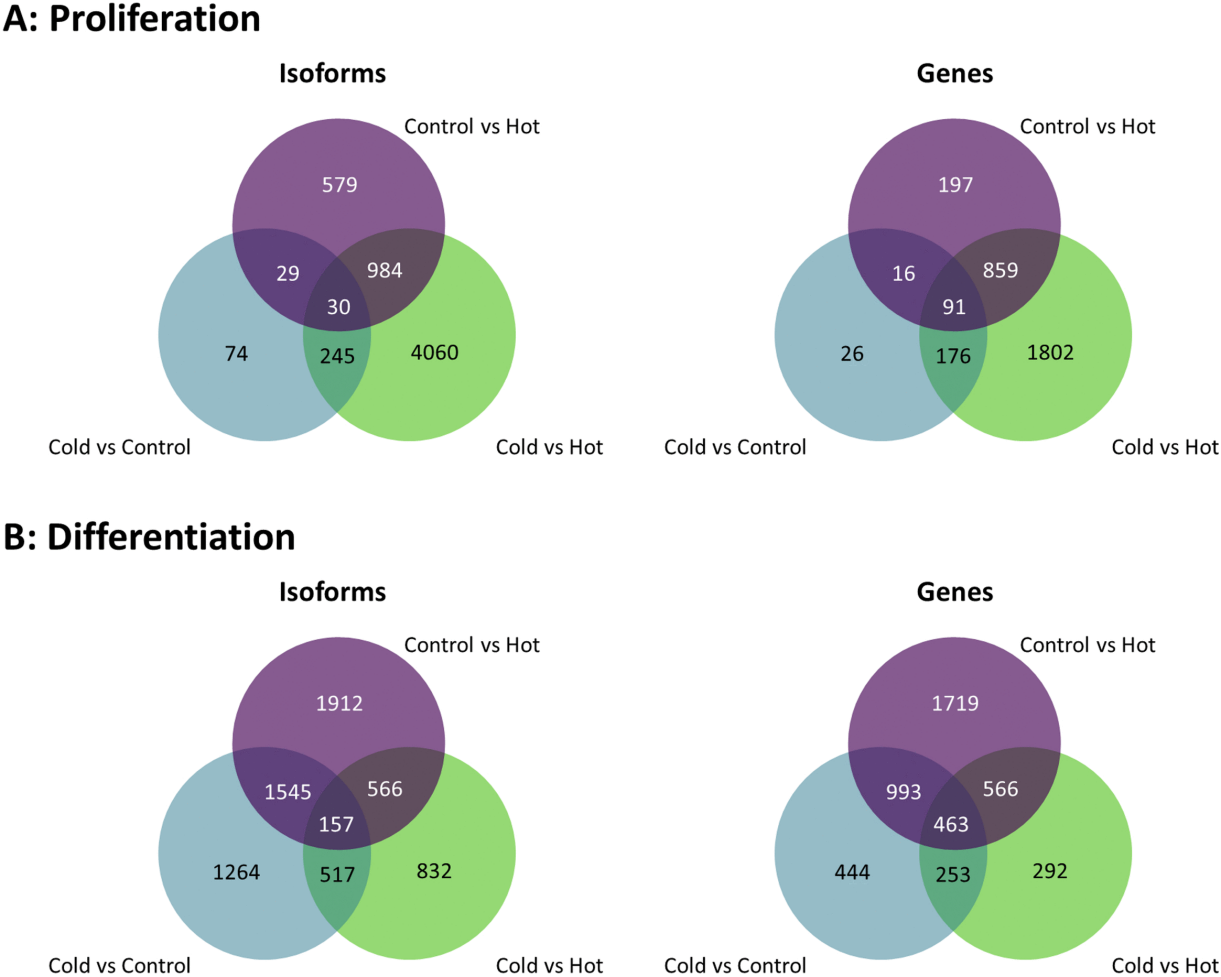
Venn diagrams showing the distribution of transcript isoforms and number of AS genes identified in the treatment comparisons in proliferating (A) and differentiating (B) SCs.

A total of 4,208 combined genes was observed to have at least one isoform switching event with 91 being alternatively spliced across all thermal comparisons (33°C vs 38°C vs 43°C) at the proliferation stage and 463 genes alternatively spliced across all thermal comparisons in the differentiation stage (Fig 2). A list of all predicted IS events (adjusted p-value < 0.01) can be found in Table S2 in S1 File. ISA dIF scores ranged from 1 to −1 with an average of 0.00044 and a sample standard deviation of 0.279.

In the proliferating SCs, 6,001 unique ISEs were predicted from a total 7,319 events involving 3,167 genes. Of the total ISEs, 1,622 occurred in heat-treated SCs (Fig 2A) and 378 in the cold treatment, with 59 ISEs were shared between the two comparisons. A total of 5,319 ISEs was observed in comparison of cells at the two temperature extremes (33°C vs 43°C) with 4,060 (involving 1,802 genes) being unique (Table S2 in S1 File and Fig 2A).

In the differentiating SCs, 6,793 unique ISEs were predicted from a total 9,735 observed events involving 3,486 genes. Of the total ISEs, 4,180 occurred in heat-treated SCs (Fig 2B) and 3,483 in the cold-treated SCs, with 1,702 ISEs shared between comparisons. A total of 2,072 ISEs was observed in comparison of cells at the two temperature extremes (33°C vs 43°C) with 832 (involving 292 genes) being unique (Table S2 in S1 File and Fig 2B).

**Fig 3:**
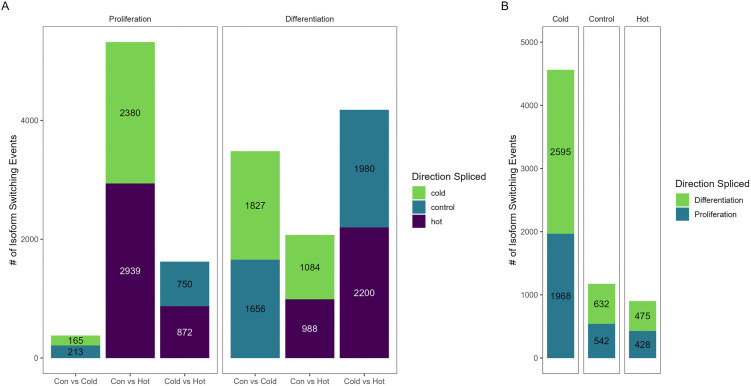
Distribution of isoform switching events in thermal treatment comparisons (A) and in cell stage comparisons (B).

The directional frequency of splicing events was fairly even across treatments. As seen in Fig 3A, direction spliced within thermal treatment comparisons are within 10% of each other. In proliferating SCs, the number of splicing events observed (%) was 165 (43.5%) vs 213 (56.5%) in the cold treatment vs the control treatment, 872 (53.8%) vs 750 (46.2%) in the heat treatment vs control treatment and 2380 (44.7%) vs 2939 (55.3%) in the cold vs heat treatment comparison. In differentiating SCs, direction of splicing showed similar frequency rates with 1827 (52.5%) vs 1656 (47.5%) in the cold treatment vs control treatment, 2200 (52.6%) vs 1980 (47.4%) in the heat treatment vs control treatment; and 1084 (52.3%) vs 988 (47.7%) in the cold treatment vs heat treatment comparison (Fig 3A).

Functional enrichment analysis of genes from the proliferating cells in DAVID found 2 annotation clusters significantly enriched in the DASs from the control vs cold comparison (Table S3 in S1 File). Highest enrichment score (1.068) occurred in cluster 1 containing the category GO:0046872-*metal ion binding* (28 genes, p = 0.0095). Analysis of the control vs hot comparison found 14 clusters with significant enrichment (p < 0.05) (Table S3 in S1 File). The top 5 clusters (enrichment score 1.470–2.462) included categories with the terms GO:0004197 *cysteine-type endopeptidase activity* (10 genes, p = 0.0024), GO:0035904 *aorta development* (5 genes, p = 0.0015), GO:0036444 *calcium import into the mitochondrion* (4 genes, p = 0.0111), GO:0005524 *ATP binding* (117 genes, p = 0.0015) and GO:0046872 *metal ion binding* (86 gene, p = 0.0123). Due to the increased number of DASs identified in the differentiating cell comparisons, a higher number of clusters was identified in the enrichment analysis. In the control vs cold comparison, 37 significant clusters (p < 0.05) were identified in DAVID. The top 5 clusters (enrichment score 1.227–4.410) included categories with the terms GO:0005643 *nuclear pore* (11 genes, p = 0.00002), GO:0046872 *metal ion binding* (176 genes, p = 0.000014), GO:0031941 *filamentous actin* (6 genes, p = 0.0105), and GO:0030892 *mitotic cohesin complex* (3 genes, p = .0.02223). The control vs hot comparison identified 32 significant clusters. Included in the top 5 clusters (enrichment score 1.108–4.006) were categories with the terms GO:0016579 *protein deubiquitination* (26 genes, p = 0.000002), GO:0046872 *metal ion binding* (192 genes, p = 0.00013), GO:0017056 *structural constituent of* *nuclear pore* (10 genes, p = 0.0005), GO:0031941 *filamentous actin* (6 genes, p = 0.0188), and GO:0000030 *mannosyltransferase activity* (5 genes, p = 0.044317).

**Fig 4:**
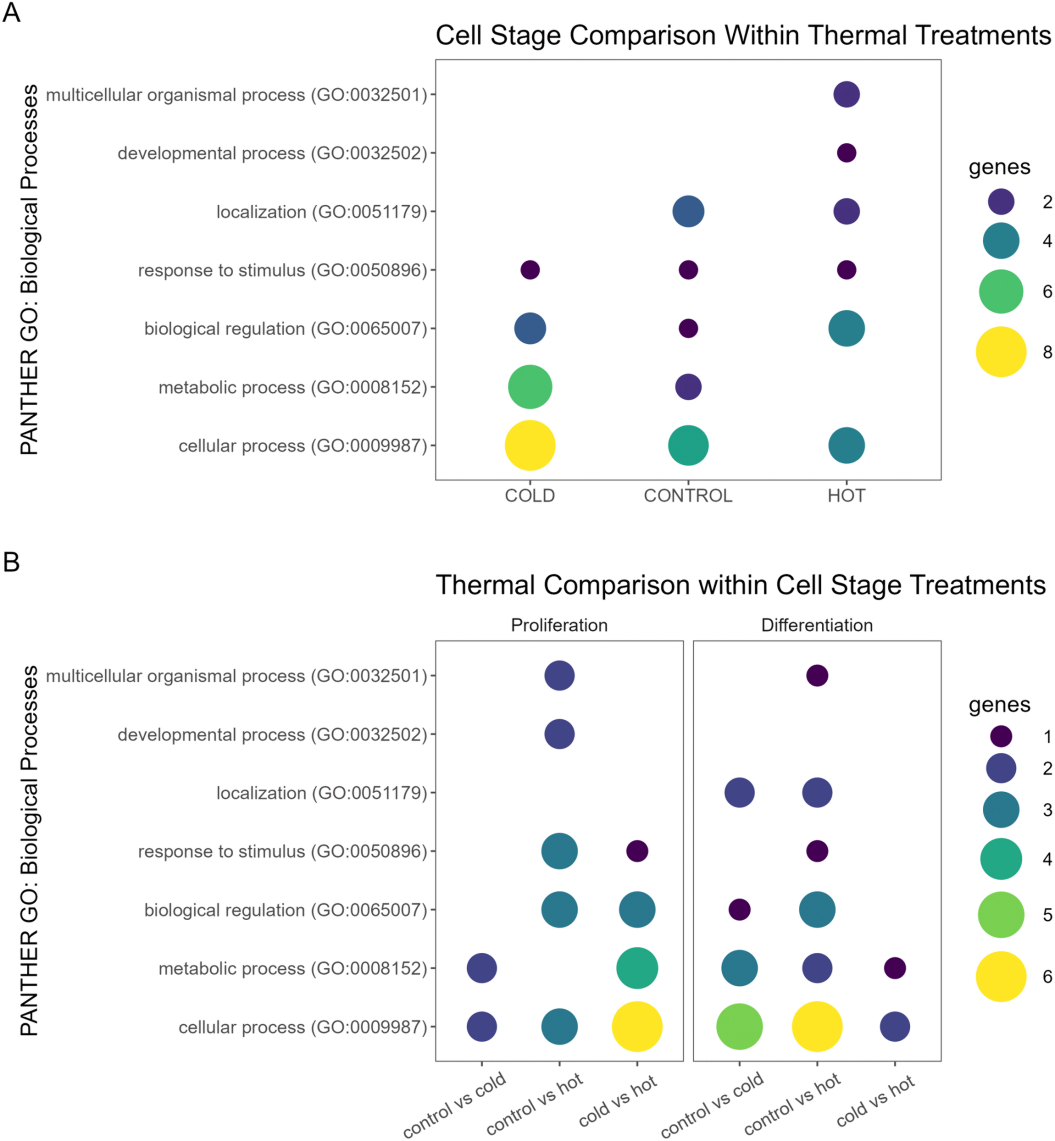
Bubble plot of differentially alternatively spliced genes (DASs) associated with GO: Biological Process categories in (A) comparisons of proliferating and differentiating SCs by treatment temperature and (B) thermal comparisons of SCs by cell stage.

To highlight common biological processes most affected by AS, GO pathways of the genes comprising the top 25 isoform switching events (Table S4 in S1 File) were examined with PANTHER. Of the seven processes shared among all comparisons, GO:0009987 *cellular* process, GO:0008152, *metabolic processes*, and GO:0065007 *biological regulation* were most represented as being potentially affected by AS due to thermal treatments within cell stages (Fig 4A; Table S5 in S1 File). Because the PANTHER functional enrichment analysis was restricted only to the top 25 events, the scope of the identified biological pathways be narrow.

### Cell stage comparisons

A total of 2,800 genes was observed to have at least one isoform switching event in cell stage comparisons (proliferation vs differentiation) within temperature, with a similar distribution by event type across the three temperature treatments (Table 1, Fig 3B). The cold treatment (33°C) comparison of proliferating and differentiating cells had the greatest number of AS events (n = 14,475) and isoform switches (n = 4,563) followed by the control treatment (38°C), where 9,538 significant AS events and 1,174 isoform switches were observed (Table S2 in S1 File). The fewest number of events occurred between proliferation and differentiation in the heat-treated cells (43°C), with 2,691 AS and 903 isoform switches observed. The frequency of up splicing was higher for differentiating SCs at all temperatures (0.569, 0.496 and 0.526 in cold, control and hot, respectively). GO pathways were also identified in PANTHER for the top 25 isoform switching events for the cell stage comparisons (Table S5 in S1 File, Fig 4B). Here the processes that were most likely to be impacted by AS across cell stage within all thermal treatments were also GO:0009987 *cellular process,* GO:0008152, *metabolic process*, and GO:0065007 *biological regulation*.

### DAS-DEG comparisons

**Fig 5:**
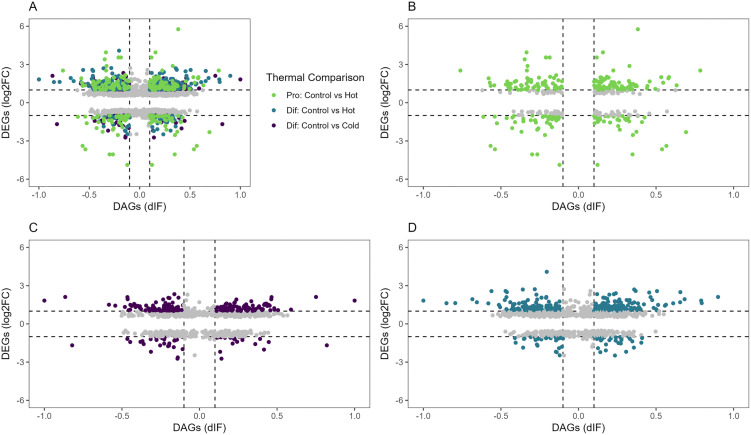
Relationship between differentially alternatively splice genes (DASs) and differentially expressed genes (DEGs) by thermal comparison: Proliferation: Control vs Hot (B); Differentiation: Control vs Cold (C); Differentiation: Control vs Hot (D) and all thermal comparisons (A).

We next investigated possible association between the observed differences in mRNA splicing (as determined by the number of AS events) and overall gene expression. The number of AS isoforms and ISEs could also be influenced by ascertainment bias, where the number of low frequency AS events is limited by the number of RNA reads in the data set. To test for association between differentially spliced genes (DASs) and differentially expressed genes (DEGs) statistically significant DASs (padj < 0.01) and DEGs (FDR < 0.01) were plotted by changes in expression (dIF for DASs and log_2_FC for DEGs) (Fig 5; Table S6 in S1 File). As seen in the comparisons, distribution appears to be skewed towards DEGs with higher |Log_2_FC|. Chi-square tests were performed to test significant differences, and all comparisons rejected the null hypothesis of random distribution of splicing and gene expression (Table S7 in S1 File).

**Fig 6:**
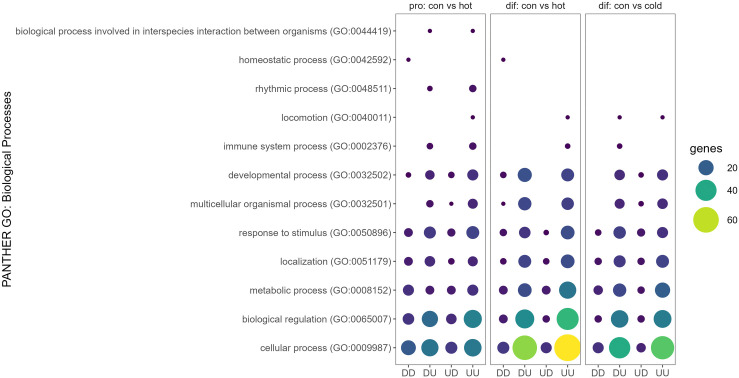
Bubble plot of genes that are both differentially expressed and differentially spliced associated with GO: Biological Process categories in temperature comparisons of proliferating and differentiating SCs. Genes are grouped by directionality of expression and splicing: downspliced-downregulated (DD), downspliced-upregulated (DU), upspliced-downregulated (UD), upspliced-upregulated (UU).

A GO Pathway analysis was also conducted in PANTHER to see if directionality of splicing/expression could be associated with specific GO pathways. Subsets of genes that fell into four categories were compared including, down regulated and down-spliced (DD), down regulated and up-spliced (DU), upregulated and down-spliced (UD), and up regulated and up-spliced (UU). Significant GO pathways shared by at least two comparisons are presented in Fig 6. As shown, the GO categories *cellular process* (GO 000987) and *biological regulation* (GO 0065007) are represented by the greatest number of genes with a larger effect seen for the differentiating SCs.

### Functional effects of isoform variation

Genes identified in the turkey SCs uniquely demonstrate potential functional effects of AS. Two such examples are Brain-Derived Neurotrophic Factor (*BDNF*) and the PDZ and LIM domain 5 protein (*PDLIM5)*. Two *BDNF* isoforms (ENSMGAT00000017275; MSTRG.17092.1) were predicted in the turkey SCs. Isoform ENSMGAT00000017275 (792 bp) appears in the hot thermal treatments in both the Proliferation: Cold vs Hot, and the Proliferation: Control vs Hot comparisons and in both the proliferation and differentiation cell stage comparisons Hot: Pro vs Dif. Isoform MSTRG.17092.1 (986 bp) appears to be differentially spliced in both the cold and hot thermal treatments in the Proliferation: Cold vs Hot comparison (33°C vs 43°C). Two AS events differentiate these two isoforms. Isoform ENSMGAT00000017275 has an alternative transcription start site and isoform MSTRG.17092.1 has two regions of mutually exclusive exons and these potentially differ by 17 aa (263 vs 246 aa, respectively), depending on translational start.

**Fig 7:**
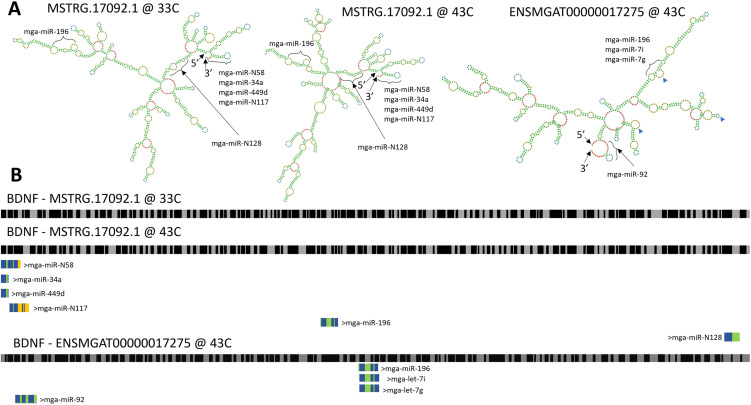
Predicted secondary fold structures for two observed isoforms of *BDNF.* **(A)** RNA folding plots of two isoforms with observed differential splicing. **(B)** Diagram depicting accessible and non-accessible sites along a linear mRNA molecule. Black bars indicate unpaired nucleotides; grey bars indicate paired nucleotides. Location of miRNA binding sites is as predicted by MiRanda 2.0 [40]. Color of miRNA denotes miRNA pairing probability: green = region can pair with transcript; yellow = region can pair with transcript at one of the predicted temperatures; blue = region cannot pair with transcript.

RNA folding predictions (Fig 7a) demonstrate how temperature and AS events can result in a modified secondary structure in *BDNF* that could have consequences for miRNA-RNA interactions and downstream translation. Potential interactions of the *BDNF* isoforms with miRNAs found to be expressed in SCs [30] are shown in Fig 7b. Since the isoforms are generated from alternate transcription start sites, the resulting RNA molecules have different 5’UTR sequences, and when folded, have different 5’ and 3’ accessibilities.

**Fig 8:**
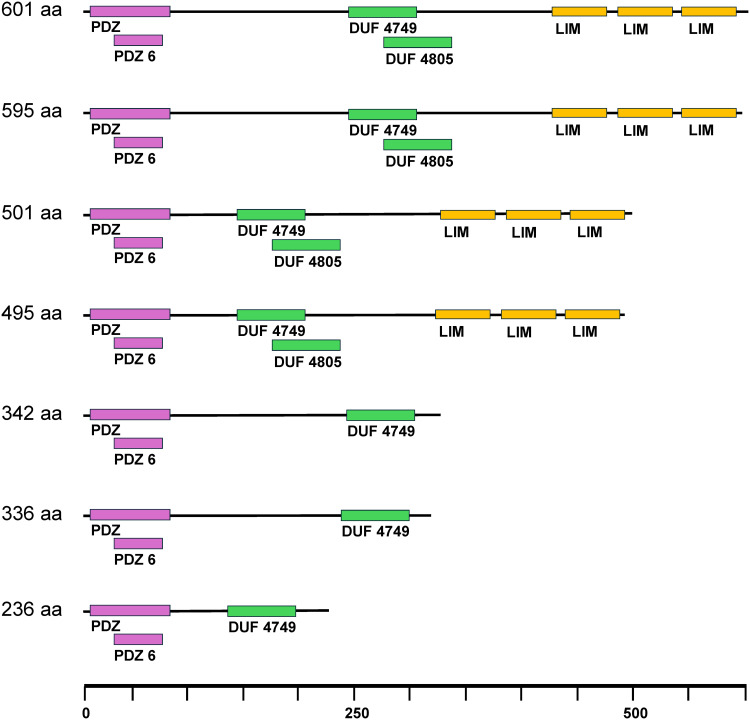
Illustration of PDLIM5 isoforms predicted in the turkey SCs. PDZ domains are denoted in purple, LIM domains in yellow and domains of unknown function in green.

PDLIM5 is a member of a family of proteins defined by presence of a PDZ domain at the N terminus and one to three LIM domains at the C-terminus. Ten isoforms of *PDLIM5* were identified in the turkey SCs (Table S2 in S1 File). Length variants included 4 “long isoform” putative proteins with 3 LIM domains (coding regions ranging from 495 to 601 aa) and 3 “short isoform” putative proteins ranging (236–342 aa) that lacked the LIM domains (Fig 8). All isoforms contained the PDZ domain and one or two domains of unknown function located between the PDZ and LIM domains. Six isoforms were involved in 19 significant IS events in the turkey SCs (Table S8 in S1 File). Although a distinct splicing pattern for PDLIM5 was not fully resolved in our experimental system, general trends were observed. Short isoforms (242 aa; 336 aa) were more abundant in the control and hot treatments during the cold vs. hot (proliferation; differentiation) and cold vs. control (differentiation) comparisons. In contrast, long isoforms (495 aa; 595 aa) were more abundant in the cold treatments for those same comparisons. Additionally, the 342 aa and 505 aa isoforms increased in frequency in differentiating SCs under control and hot conditions, whereas the 495 aa isoform increased in frequency in proliferating SCs under those same conditions.

## Discussion

### Alternative splicing and muscle SCs

Cultured muscle SCs represent an ideal system for delineating alternative splicing mechanisms during myogenesis. We have used turkey skeletal muscle SCs to examine aspects of gene regulation in response to thermal stress. Our transcriptome studies have utilized RNAseq data to examine gene expression [(mRNA transcription, 24,25,27,28)], non-coding RNAs [(miRNA, 30)], post-transcriptional modification [(circRNAs, 41)] and potential RNA/RNA interactions. This study focused on a major contributor to transcript diversity, AS that plays a significant role in generating proteomic diversity necessary in SC function during muscle growth and regeneration. Comparison of the diverse RNA types identified through RNA sequencing to whole genome sequence annotations highlights the significant role of AS in transcriptome and ultimately proteome diversification [42, 43]. Our analysis of AS in skeletal muscle SCs found evidence for splicing variants in over 5000 expressed genes with over 23,000 isoform switching events among the experimental groups in response to temperature.

Splicing of pre-mRNAs is controlled by the spliceosome complex that defines and controls the inclusion/exclusion of RNA sequence (exons and introns) in the final mRNA [44]. The process is controlled by specific cis-acting sequence elements within the pre-mRNA that are recognized by the RNA-binding proteins of the spliceosome and function as enhancer or silencers [45]. RNA-binding proteins are themselves regulated by signal transduction pathways that control their expression or activity. Interactions between pre-mRNA and the splicing machinery are also influenced by RNA secondary structure and RNA-RNA interactions [46]. Alternative transcription start and termination site usage were prominent in the turkey SCs as were exon-skipping (ES) events. ES events are the most frequent AS event in mammals, with the majority involving a single exon [47], and with tissue-specific splicing events being high in skeletal muscle [48].

Categorizing AS events within select tissues can identify novel mechanisms of gene regulation. For example, genes with the highest number of AS events in the turkey SCs were *NCAM1*, a cell adhesion molecule important in the development and plasticity of neuromuscular junctions [49], *R3HDM1*, a gene required in mice for SC proliferation and differentiation [50], and supervillin, a costameric protein that contributes to myogenic membrane structure and differentiation [51]. In humans, multiple isoforms of NCAM have been described and a secreted form of the protein is produced in muscle and brain through AS [52]. Supervillin also has multiple transcript variants encoding different isoforms [53].

Skeletal muscle is known to have a high number of tissue-specific events [48]. In their review of AS in muscle, Nakka et al. [54] identified 35 genes differentially spliced during myogenesis. Orthologs of at least 24 of these were identified as AS in the turkey SCs in the present study. Analysis of high-throughput data has identified cis-regulatory elements and trans-acting splicing factors that are modulated by alternative splicing in the muscle cells. For example, transcription factors PAX7 and PAX3 that are key regulators of SCs undergo AS [55], whereas the important myogenic regulatory factors (MYOD, MYF5, and Myogenin) do not. In some cases, MYOD itself is necessary for muscle-specific alternative splicing [56].

Our combined analysis of AS and differential expression found that genes with higher fold change in differential expression analysis were more likely to show significant changes in AS. One possibility for this observation is changes in the expression of genes with specific roles in RNA splicing caused by thermal challenge. Elevated temperature can significantly affect the gene splicing profile and therefore AS is considered as an integral part of a stress response. This hypothesis is further supported by studies in other animals. Under heat stress, AS events in rats increased by 20% [57], and in catfish and bass, thermal stress increased both AS events (29%) and AS genes (25.8%) [12, 58].

In our original RNAseq studies [25, 28], several genes with predicted roles in RNA splicing were identified as being differentially expressed but with low fold change differences. For example, in heat-treated SCs, 14 splicing factors were significantly down regulated, with four, *ERH* (ERH mRNA splicing and mitosis factor), *SRSF3*, *SRSF5* (serine and arginine rich splicing factors 3 and 5), and *PUF60* (poly (U) binding splicing factor 60), being down regulated in both proliferation and differentiation. In addition, 5 genes were upregulated in heat-treated cells including serine and arginine rich splicing factor 4 (*SRSF4*) splicing regulatory glutamic acid and lysine rich protein 1 (*SREK1*), NOVA alternative splicing regulator 1 (*NOVA1*), and two muscleblind-like splicing regulators (*MBNL1*, *MBNL2*). One of these, *MBNL2*, was significantly upregulated in both proliferating and differentiating SCs. MBNL2 is an important RNA-binding protein that functions as a regulator of alternative mRNA splicing. In humans, it is primarily expressed in skeletal muscle and the brain, and is involved in diseases like myotonic dystrophy [59]. Thus, given the significant expression changes in genes associated with splicing, it is reasonable to expect concurrent change in observed splicing events.

It is important to note that some AS events are likely not detected in our RNAseq data. For example, low frequency AS events have an increased likelihood of being detected for genes with higher expression. The average number of sequence reads per library in the original RNAseq experiments was 22.7M in proliferating cells [25] and 22.8M in differentiating cells [28]. While this is adequate for surveying expression in cell culture-based experiments, it may not be ideal for capturing all splice variants in our retrospective analysis.

In addition to diversifying protein function, AS has the potential to generate mRNA isoforms that include premature stop codons resulting from frameshifts that may be targeted for nonsense-mediated decay (NMD). The location of a premature termination codon (PTC) relative to the canonical stop site typically determines the consequences of such variants [60]. If the PTC occurs upstream of the final exon-exon junction, it often triggers NMD resulting in a loss of protein expression or significantly reduced levels. If the PTC is close to the natural stop codon or within the final exon, the transcript may evade NMD resulting in a non-functional protein or one with altered function.

Prior investigations of the turkey ryanodine receptors by members of our group, identified splice variants in *RYR3* with alternative predicted outcomes. One variant, AS-193 with an introduced frameshift, likely generates a truncated polypeptide that may be targeted for NMD. In contrast, the turkey AS-81 variant with a deletion of 27 amino acids, maintained the reading frame Chiang et al. [61] producing a truncated protein. A similar *RYR3* variant in humans (AS-8a) lacks a 29-amino acid segment encompassing a putative transmembrane helix, but can still participate in heterotetramer formation with wild-type *RYR3*, ultimately suppressing its function and reducing caffeine sensitivity [62]. AS-8a also forms heteromers with RYR2 suppressing its activity. This suggests that the truncated turkey variant may also alter RYR function. The examples of *RYR3* highlight the variable outcomes of splicing events where AS could similarly lead to non-functional proteins targeted for degradation or frameshifted proteins.

### Implications for isoform variation

Differential RNA splicing in response to environmental cues emphasizes the role of alternative isoforms in cellular responses. Alternative gene splicing leads to structural changes in mRNA that expands the range of proteins a single gene can encode. Structural changes in proteins change binding properties, subcellular localization, protein stability, and ultimately protein function [15].

Modifications to mRNA not only have implications for the coding sequence, but can alter folding (secondary and tertiary structure), stability, and mRNA interactions with other RNAs and proteins, including the translational machinery. Site accessibility is critical in determining mRNA-miRNA interactions and mutations that reduce accessibility can have comparable effects on translation as changes in target sequence complementarity [63]. Changes in miRNA interaction could affect downstream translational regulation. Studies also suggest that the amino acids encoded by alternative exons often occur on the surface of proteins or in unstructured regions and may not change core structure [64].

As highlighted, isoforms of *BDNF* potentially differ in RNA folding and non-coding RNA interactions that may also be affected by temperature. BDNF is of particular interest due to its unique transcription in humans where multiple transcripts are generated from nine functional promoters to create mRNAs that all encode for the same mature BDNF protein [65]. BDNF is best known as a key molecule for neuronal plasticity and neurogenesis, playing a crucial role in the brain [66]. BDNF activation in chickens is critical in thermal-experience-dependent brain plasticity and expression is induced during thermal conditioning [7]. The biological relevance of BDNF in satellite cell biology centers primarily on its role as a local regulator of muscle regeneration and repair. BDNF is also produced in skeletal muscle and plays a role in muscle metabolism, regeneration, and neuromuscular junction support. The gene functions in muscle development as a myokine, in metabolism to enhance lipid oxidation by activating AMP-activated protein kinase (AMPK) and in myogenic differentiation and muscle regeneration. In rats, it is expressed in skeletal muscle SCs where it may act to inhibit myogenic differentiation and thereby helping to maintain the population of muscle progenitors in adult muscle [67, 68]. In chickens, novel AS variants of BDNF are described with apparent differing levels of DNA methylation [69].

*PDLIM5* exemplifies the crucial role that AS plays in determining the presence and architecture of functional domains within proteins. Splicing of the primary *PDLIM5* transcript generates multiple variants that encode isoforms differing in length and presence/absence of LIM domains. Functionally, PDLIM5 binds to cytoskeleton and membrane proteins through its PDZ domain and interacts with signaling molecules, protein kinases and transcription factors, through its LIM domain [70]. Cytoskeletal interaction includes binding proteins like α-actinin and PDLIM5 is found in the Z-disk of sarcomeres. A study of chicken skeletal muscle SCs suggests that PDLIM5 plays an active role in proliferation and differentiation by activating the p38-MAPK signaling pathway and in reducing the expression of muscle-development-related genes [71]. Although this study reported changes in proliferation and differentiation of chicken SCs after interfering and overexpressing *PDLIM5*, they did not address the possible role of variant isoforms.

Change in the ratio of *PDLIM5* isoforms in response to thermal challenge could directly influence myoblast proliferation and differentiation, potentially impacting muscle growth and regeneration [72]. Patterns of AS and regulatory mechanisms are conserved across species and developmental stages indicating, maintenance by natural selection and the functional importance of specific AS events [73]. In humans, the long *PDLIM* isoform (ENH1) is widely expressed in various tissues and contains three LIM domains at its C-terminus and the short isoforms are mainly expressed in cardiac and skeletal muscle and lack LIM domains [74]. Studies in rodents indicate a developmental pattern in splicing during muscle development [75] and suggest that the main effect of isoform 1 on muscle cells is to stimulate the transcription of MyoD- and/or myogenin-encoding genes [76]. PDLIM5 isoform 1 knockouts have decreased MyoD and myogenin expression whereas overexpression prevents ventricular cardiomyocyte hypertrophy [77]. Other splicing isoforms exert various effects on the development of heart and skeletal muscle by altering expression of myogenic genes and subsequently myotube formation. A study in pigs found the short form of PDLIM5 negatively regulates myoblast proliferation and differentiation [72]. These AS responses to thermal challenge demonstrate the broader potential effects of temperature change in SCs and possible downstream impacts on developing muscle.

For example, alternative splicing provides the transcriptomic flexibility required for SCs to transition between states of dormancy, division, and maturation. During the shift from quiescence to proliferation, the splicing of cell-cycle regulators and signaling receptors, sensitizes the cell to growth factors, stimulating the SC to move toward differentiation. Furthermore, AS of key fate-determinants helps control SC asymmetric division, that ensures a balance between new muscle cells and maintaining the stem cell reservoir. Disruption of this balance could lead to depletion of the regenerative pool (stem cell exhaustion). It is hypothesized that stem cell exhaustion may be a driver of Wooden Breast syndrome in fast-growing chickens [78–80].

Epigenetic changes, as the result of thermal challenge may also significantly influence AS events, by altering the physical landscape of DNA to dictate function of the splicing machinery. This is thought to occur primarily through the effect of thermal stress on DNA methylation or histone marking (e.g., histone H3 lysine 36 trimethylation, H3K36me3) to affect the travel speed of RNA polymerase II. H3K36me3 is an epigenetic mark associated with active gene transcription. Sudden shifts in temperature can cause RNA polymerase to either stall, allowing recognition and inclusion of “weaker” exons, or accelerated, leading to exon skipping [81, 82]. Epigenetic marks can also act to directly recruit specific splicing factors to a gene during the stress response. Because these modifications can persist after the temperature returns to baseline, even an acute exposure can create a lasting shift in production of protein isoforms. For example, thermal manipulation during the critical developmental window (d7 to d16 of embryogenesis) can permanently alter gene splicing of adult chickens [83]. Here, thermally-treated birds exhibited a significantly different AS response when heat challenged as adults, while appearing similar to control birds under normal conditions.

## Conclusion

This study identified thousands of genes with predicted AS events, many of which significantly responded to thermal challenge in turkey skeletal muscle SCs. The presence of alternate transcripts and their translation into protein isoforms would translate into functional differences that would likely affect the proliferation and differentiation of SCs. We anticipate that some isoform predictions are affected by persistent assembly errors in the turkey reference genome. Because identification of the AS genes and isoform switching events are based on in silico analysis, confirmatory individual gene experiments (e.g., PCR amplification) and supporting long-read RNA sequencing are needed to fully understand differential splicing and the potential effects on function. The recent availability of the chicken gene atlas provides a detailed resource of AS and 3’-untranslated region alternative polyadenylation across 28 tissues (including skeletal muscle) that will be valuable for comparative studies [84]. This present study identified a suite of DAS genes in the turkey providing both comparative data as well as hypotheses for future research on the significance of AS on post-transcriptional RNA interactions in muscle stem cells. Additional studies are needed to more fully understand how these changes in the SC mRNA pool affect poultry muscle biology and the role of AS in thermal stress response.

## Supporting information

S1 File This article contains supporting information. **Powell_SupportingInformation.xlsx** Workbook containing Supplementary Tables (S1-S8).
(XLSX)
